# Stacking effects in van der Waals heterostructures of blueP and Janus XYO (X = Ti, Zr, Hf: Y = S, Se) monolayers†

**DOI:** 10.1039/d0ra10827h

**Published:** 2021-03-25

**Authors:** Qaisar Alam, M. Idrees, S. Muhammad, Chuong V. Nguyen, M. Shafiq, Y. Saeed, H. U. Din, B. Amin

**Affiliations:** Department of Physics, Hazara University Mansehra 21300 Pakistan; Department of Materials Science and Engineering, Le Quy Don Technical University Hanoi Vietnam; Department of Physics, Abbottabad University of Science and Technology Abbottabad Pakistan binukhn@gmail.com

## Abstract

Using first-principles calculations, the geometry, electronic structure, optical and photocatalytic performance of blueP and XYO (X = Ti, Zr, Hf; Y = S, Se) monolayers and their corresponding van der Waal heterostructures in three possible stacking patterns, are investigated. BlueP and XYO (X = Ti, Zr, Hf; Y = S, Se) monolayers are indirect bandgap semiconductors. A tensile strain of 8(10)% leads to TiSeO(ZrSeO) monolayers transitioning to a direct bandgap of 1.30(1.61) eV. The calculated binding energy and AIMD simulation show that unstrained(strained) blueP and XYO (X = Ti, Zr, Hf; Y = S, Se) monolayers and their heterostructures are thermodynamically stable. Similar to the corresponding monolayers, blueP-XYO (X = Ti, Zr, Hf: Y = S, Se) vdW heterostructures in three possible stacking patterns are indirect bandgap semiconductors with staggered band alignment, except blueP-TiSeO vdW heterostructure, which signifies straddling band alignment. Absorption spectra show that optical transitions are dominated by excitons for blueP and XYO (X = Ti, Zr, Hf; Y = S, Se) monolayers and the corresponding vdW heterostructures. Both *E*_VB_ and *E*_CB_ in TiSO, ZrSO, ZrSeO and HfSO monolayers achieve energetically favorable positions, and therefore, are suitable for water splitting at pH = 0, while TiSeO and HfSeO monolayers showed good response for reduction and fail to oxidise water. All studied vdW heterostructures also show good response to any produced O_2_, while specific stacking reduces H^+^ to H_2_.

## Abstract 2

Using first-principles calculations, the geometry, electronic structure, optical and photocatalytic performance of blueP and XYO (X = Ti, Zr, Hf; Y = S, Se) monolayers and their corresponding van der Waal heterostructures in three possible stacking patterns, are investigated.

## Graphical abstract

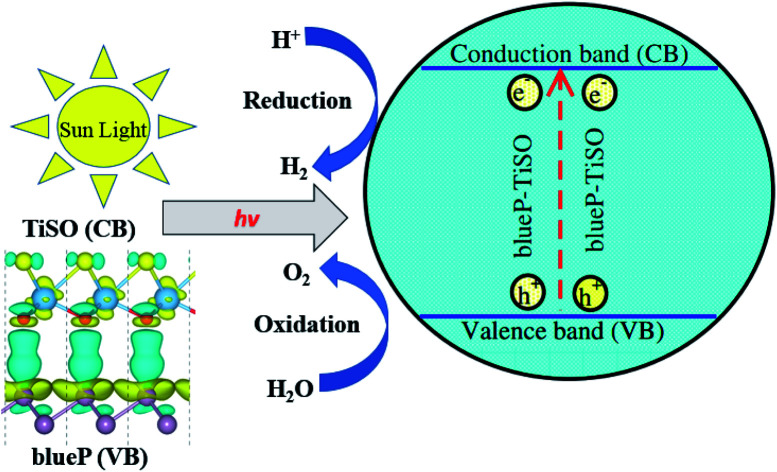

## 1. Introduction

In the family of two dimensional (2D) materials,^1–5^ transition metal dichalcogenides (TMDs) monolayers (MX_2_, M: a transition metal atom, X: a chalcogen atom), have received specific attention due to their easy preparation and multiple characteristics.^6^ Strong excitonic effects with high exciton binding energies,^7^ result in a very fast recombination rate of photongenerated electron and hole carriers, hence leading to low quantum efficiency in these materials, *i.e.* in the 10^−4^ to 10^−2^ range.^8–10^ Therefore, great efforts have been paid to upgrade the physical and chemical properties of 2D TMDs.

A new class of TMDs (MX_2_) monolayers, namely; Janus TMDs (MXY or XMY (M = Mo,W; (X ≠ Y) = S, Se, Te)), have been synthesized by chemical vapor deposition of Se(S) in MoS_2_(MoSe_2_).^11,12^ Furthermore, electronic structures and Raman vibration modes of these materials have also been associated using density functional theory (DFT) calculations.^12^ These materials are favourable for futuristic spintronic devices due to the larger SOC-induced Rashba spin splitting.^13,14^ In the family of Janus TMDs, XYO (X = Ti, Zr, Hf; Y = S, Se) monolayers are also found to be stable and much easier to achieve experimentally with promising applications in photocatalysis, nanoscale electronics and mechanical sensors.^15^

Similar to the control of dimensionality^16^ and composition,^17^ stacking *via* van der Waals (vdW)^18^ interactions is also an effective approach to tune the properties of a material^19,20^ and to design viable electronic devices.^21,22^ For instance, using DFT calculations, SiC/TMDs,^23^ MoS_2_/Si,^24^ graphene/MoSeS,^25^ Janus-MoSeTe/X(OH)_2_ (X = Ca, Mg),^26^ SnSe_2_/MoS_2_,^27^ GeC-MSSe (M = Mo, W),^28^ graphene/WSeTe,^29^ graphene/Ga_2_SSe ^30^ and TMDs/ZnO ^31^ have already been investigated in detail. Many blueP-based vdW heterostructures, such as blueP/graphene and blueP/g-GaN,^32^ blueP/BlackP,^33^ blueP/TMDCs,^34,35^ blueP/graphene,^36^ blueP/AlN ^37^ and blueP/Mg(OH)_2_,^38^ have also been fabricated and investigated.

In this framework, hexagonal symmetry and lattice mismatch of blueP with XYO (X = Ti, Zr, Hf: Y = S, Se) monolayers realize the fabrication of blueP-XYO vdW heterostructures.^15,39^ Single layer blue phosphorus (blueP) with buckled honeycomb lattice symmetry has already been successfully synthesized on a Au(111) substrate,^40^ and demonstrated theoretically as a new 2D allotrope of phosphorous poly-types.^41^ This material gained a great deal of interest in nano-electronics and in rechargeable Li-ion batteries based on the bandgap engineering and high charge capacities.^42^

Although, a new family of Janus TMDs, XYO (X = Ti, Zr, Hf: Y = S, Se) monolayers in the distorted 1T-phase, have already been investigated in detail for their versatile applications. In this respect, it is surprising that so far no investigation has addressed the 2H phase of XYO (X = Ti, Zr, Hf: Y = S, Se) monolayers. To overcome this gap and motivated by findings of the numerous vdW heterostructures discussed above, in the present study, we have systematically investigated the stabilities, electronic structures, interlayer charge transfer, optical and photocatalytic performance of the blueP and novel XYO (X = Ti, Zr, Hf: Y = S, Se) monolayers in the 2H phase and their vdW heterostructures in three possible stacking configurations.

## 2. Computational details

DFT ^43^ calculations are performed in VASP,^44,45^ with PBE ^46^ and HSE06 ^47^ functionals to describe the electron exchange and correlation energy, while the DFT-D2 method of Grimme,^48^ is adopted for vdW correction. Energy cutoff of the plane wave is set to 500 eV, criterion for the force(energy) convergence is set to 0.001 eV Å^−1^ (10^−5^ eV), and a vacuum of 25 Å is set in the out-of-plane direction to avoid artifacts of the periodic boundary conditions. A *K*-point mesh of 6 × 6 × 1 is used for geometric relaxation and 12 × 12 × 1 for the optimized structures. In addition, we added dipole correction (with DIPOL = 0.5 0.5 0.5) in the unit cell.

Furthermore, *ab initio* molecular dynamics simulations (AIMD) are performed through a Nose thermostat algorithm at 300 K for 6 ps with 1 fs time interval to investigate the thermal stabilities of monolayers and corresponding vdW heterostructures.^49^ We have employed the GW0 approach to solve the Bethe–Salpeter equation for the analysis of the imaginary part of the dielectric function.

Additionally, strain engineering is simulated by setting the lattice parameter in 
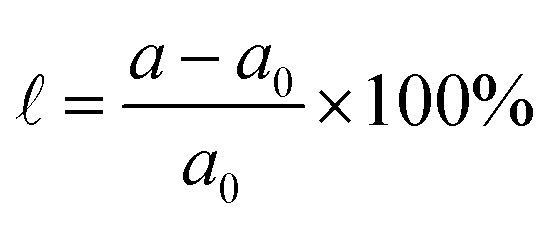
 and relaxing the atomic positions, where *a*(*a*_0_) are the strained(unstrained) lattice parameters.

## 3. Results and discussion

**Fig. 1:**
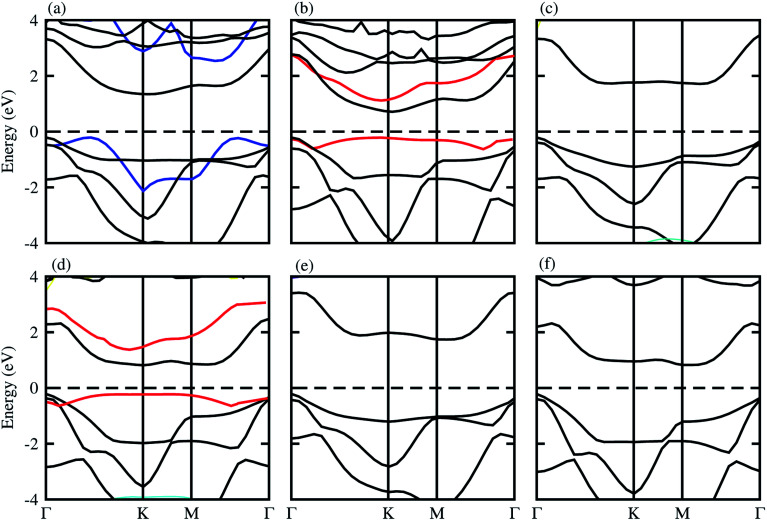
Electronic band structure of (a) TiSO, (b) TiSeO, (c) ZrSO, (d) ZrSeO, (e) HfSO, and (f) HfSeO; blue lines represent the band structure of blueP, and the red line represents strain.

The optimized lattice constant and bond length of blueP and XYO (X = Ti, Zr, Hf: Y = S, Se) monolayers are presented in Table 1, and the band structures shown in Fig. 1, they are in good agreement with available data.^50–52^ It is clear from Fig. 1, that blueP and XYO monolayers are indirect bandgap semiconductors with valence band maximums (VBM) at *Γ*-point; and conduction band minimums (CBM) at the *K*-point for TiSO, TiSeO, ZrSeO, and at the *M*–*Γ*-point for ZrSO, HfSO and HfSeO monolayers. The indirect bandgap nature of TiSO is inconsistent with the direct bandgap nature in ref. 15, while they showed that the CBM also sits at the *Γ*-point. The disagreement is due to the fact that they have used the distorted 1T-phase, while we have investigated the 2H phase of the XYO (X = Ti, Zr, Hf: Y = S, Se) monolayers.

**Table 1:** Lattice constant (*a* in Å), band length (*d*_X–Y_ and *d*_X–O_ (X = Ti, Zr, Hf; Y = S, Se) in Å) and bandgap (in eV) of blueP, TiSO, TiSeO, ZrSO, ZrSeO, HfSO, and HfSeO monolayers

Monolayers	blueP	TiSO	TiSeO	ZrSO	ZrSeO	HfSO	HfSeO
*a* (Å)	3.27	3.10	3.17	3.35	3.40	3.30	3.36
*d*_X–Y_	2.26	2.52	2.60	2.57	2.69	2.55	2.55
*d*_X–O_	—	2.10	2.03	2.14	2.14	2.14	2.19
*E*_g_	2.77	1.58	0.91	1.97	1.18	1.97	0.83

Strain engineering is an influential strategy to tune the bandgap of 2D materials.^53^ Therefore, we have also evaluated the effect of strain on the band structure of XYO (X = Ti, Zr, Hf: Y = S, Se) monolayers, see Fig. 1 (red line). Generally, compressive/tensile strain reduces/increases the X–Y(O) bond length, while it increases/reduces the coupling between the X and Y(O) atoms, hence strengthens/weakens the splitting between the bonding and antibonding states and thus modifies the bandgap accordingly.^53^ In the case of XYO (X = Ti, Zr, Hf: Y = S, Se) monolayers, tensile strain of 8(10)% leads to a transition of indirect TiSeO(ZrSeO) monolayers to a direct bandgap nature of 1.30(1.61) eV. Bandgap variation with external strain using DFT calculations has the same trend compared to the sophisticated methods like the configuration interaction method.^54^ In the case of the tensile strain, the energy of the CBM(VBM) at *K*-points reduces(rises) rapidly. The position of the VBM and CBM in BZ are fixed at the *K* point, while their corresponding energy varies with respect to the Fermi level. The opposing behaviour of increasing the bandgap value with tensile strain is due to the change in the position of the VBM and CBM *i.e.*, the indirect bandgap nature of TiSeO and ZrSeO monolayers.

**Fig. 2:**
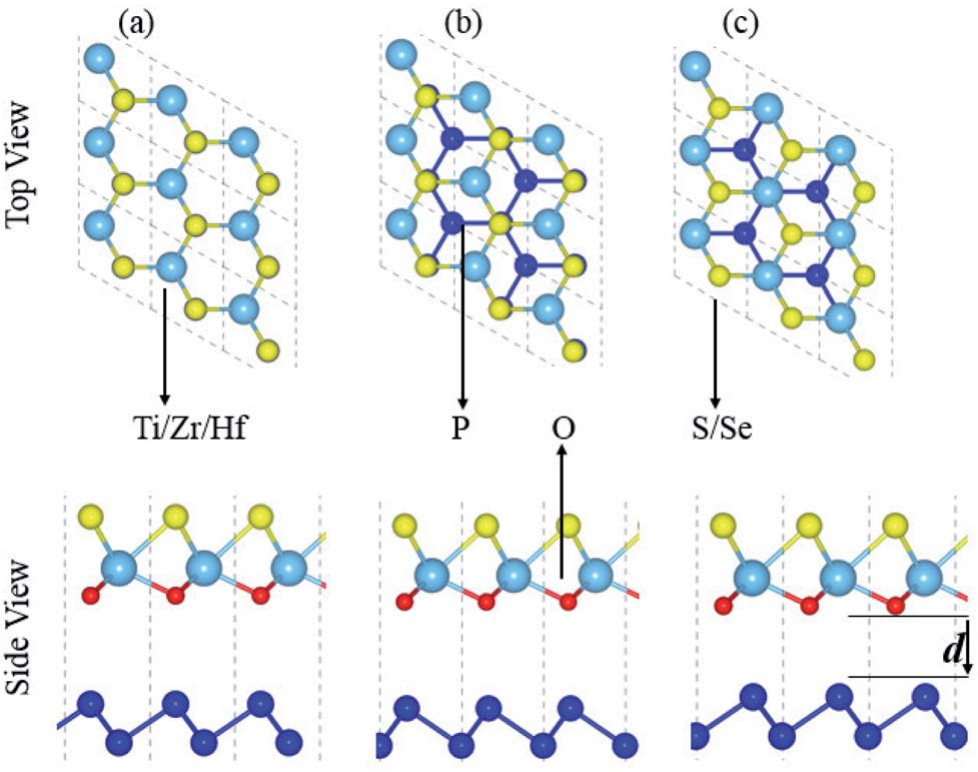
Possible stacking of blueP-XYO (X = Ti, Zr, Hf; Y = S, Se) vdW heterostructures.

Fabrication of vdW heterostructures are also realized in the case of the blueP and XYO (X = Ti, Zr, Hf; Y = S, Se) monolayers, due to hexagonal symmetry and experimentally achievable lattice mismatch. Therefore, three possible stacking patterns of the blueP and XYO (X = Ti, Zr, Hf; Y = S, Se) monolayers in the form of blueP-XYO (X = Ti, Zr, Hf; Y = S, Se) vdW heterostructures are investigated, see Fig. 2. In the case of blueP-TiSO vdW heterostructures, stacking: (a) a Ti atom of the TiSO monolayer is fixed on one P atom of the blueP monolayer, while the O and S atoms are fixed on the top of another P atom; (b) O and S atoms of the TiSO monolayer are fixed on the top of one P atom of the blueP monolayer, while Ti and another P atom are on hexagonal sides; (c) a Ti atom is placed on top of one P atom while the S (O) and other P atoms are on the hexagonal sites. Similar stacking patterns are also studied for blueP-TiSeO, blueP-ZrSO, blueP-ZrSeO, blueP-HfSO, and blueP-HfSeO vdW heterostructures.

Binding energies calculated by; *E*_b_ = *E*_blueP-XYO_ − *E*_blueP_ − *E*_XYO_, where *E*_blueP-XYO_ is the energy of the blueP–XYO vdW heterostructure and *E*_blueP_(*E*_XYO_) is the energy of the isolated blueP(XYO (X = Ti, Zr, Hf: Y = S, Se)) monolayer. The calculated negative binding energy presented in Table 2 indicates that the above three possible stacking configurations are favorable energetically and can be fabricated experimentally. More negative value of *E*_b_ means a more stable system with stronger interface binding, hence results listed in Table 2 show that stacking (c) is the most favorable stacking pattern for these vdW heterostructures. Although stacking (c) is the most favorable stacking pattern, but stacking (a) and (b) are also energetically stable and can be obtained experimentally, therefore we have further investigated all the three possible stacking patterns of the above mentioned vdW heterostructures.

**Table 2:** Lattice constant (*a* in Å), binding energies (*E*_b_ in eV), inter layer distance (*d* in Å), bandgap (*E*_g_ (HSE) in eV), work function (*ϕ* in eV) in stacking-I, stacking-II and stacking-III of blueP-XYO (X = Ti, Zr, Hf; Y = S, Se) vdW heterostructures

Heterost.	blueP-TiSO	blueP-TiSeO	blueP-ZrSO	blueP-ZrSeO	blueP-HfSO	blueP-HfSeO
*a*	3.18	3.22	3.30	3.33	3.29	2.82

**Stacking-I**						
*E*_b_	−0.439	−0.659	−0.132	−0.290	−0.075	−0.201
*d*	3.49	3.41	3.35	3.25	3.52	3.52
*E*_g_	0.94	0.97	1.84	0.99	1.94	1.04
*Φ*	2.03	2.21	2.96	2.47	2.18	2.11

**Stacking-II**						
*E*_b_	−0.437	−0.658	−0.131	−0.289	−0.072	−0.198
*d*	3.52	3.45	3.40	3.30	3.60	3.55
*E*_g_	0.91	1.01	1.93	1.03	1.90	1.01
*ϕ*	1.85	1.98	2.39	2.79	2.21	2.14

**Stacking-III**						
*E*_b_	−0.481	−0.693	−0.213	−0.382	−0.223	−0.359
*D*	3.25	3.29	3.23	3.39	3.26	2.23
*E*_g_	0.88	0.99	1.83	0.92	1.88	0.97
*ϕ*	2.00	2.13	2.04	2.73	2.12	2.04

Performing AIMD simulations^55,56^ with a time step of 1 fs and total simulation time of 6 ps, we have also investigated the thermal stabilities of the unstrained (strained) XYO (X = Ti, Zr, Hf: Y = S, Se) monolayers (see Fig. S1†) and corresponding vdW heterostructures (see Fig. S2 and S3†). Our AIMD simulation showed that the atomic structure of the studied unstrained(strained) XYO (X = Ti, Zr, Hf: Y = S, Se) monolayers and corresponding vdW heterostructures is maintained at 300 K after heating to 6 ps, with a very small difference in total energy. We have also calculated the phonon spectra for 10% tensile strain of the ZrSeO monolayer, presented in Fig. S4.† Phonon dispersion exhibits no imaginary frequency, which confirms the stability of a ZrSSe monolayer under 10% tensile strain.

**Fig. 3:**
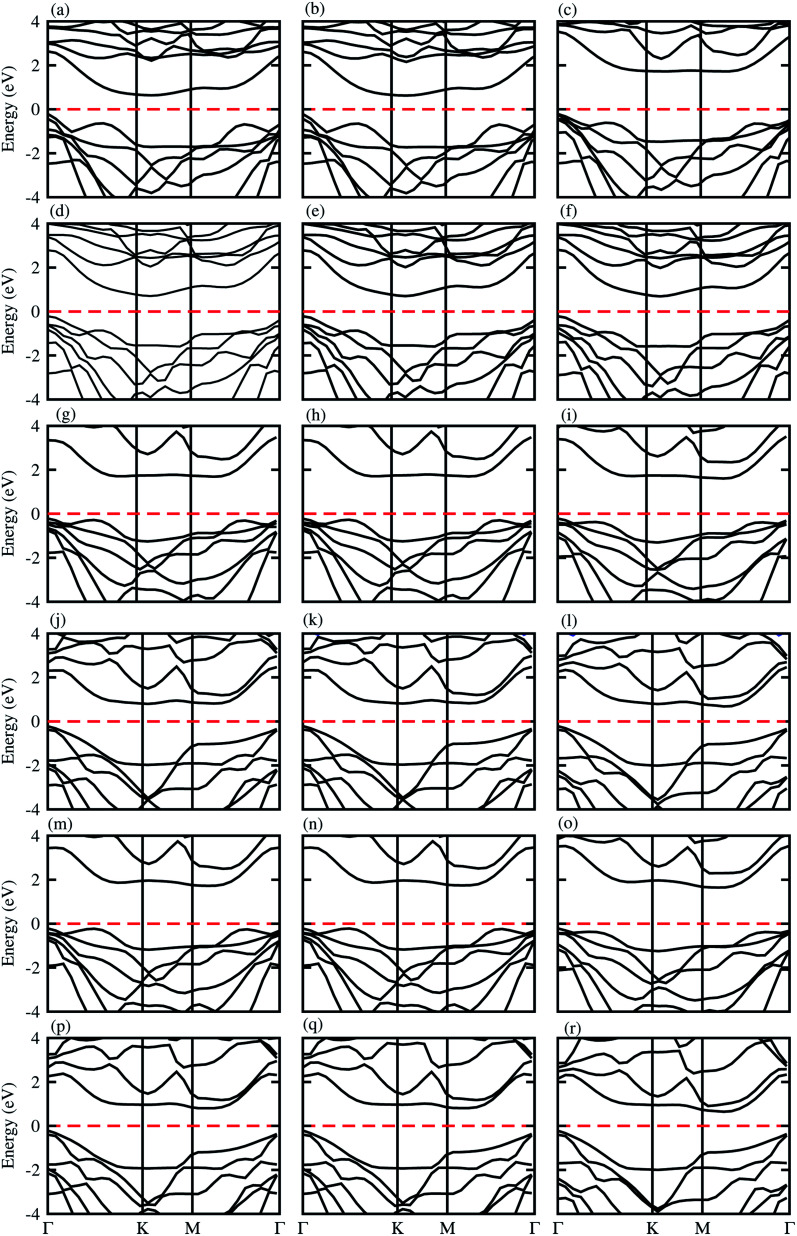
Electronic band structure of blueP-TiSO (a–c), blueP-TiSeO (d–f), blueP-ZrSO (g–i), blueP-ZrSeO (j–l), blueP-HfSO (m–o), and blueP-HfSeO (p–r), where the first column is for stacking-I, the second column is for stacking-II and the third column is for stacking-III of the corresponding vdW heterostructures.

The electronic band structures presented in Fig. 3, show that blueP-XYO (X = Ti, Zr, Hf; Y = S, Se) vdW heterostructures in three possible stacking patterns are indirect bandgap semiconductors with VBM located at the *Γ*-point of BZ; while the CBM are located at the *Γ*–*K*-point of BZ for blueP-TiSO (Fig. 3(a–c)), blueP-TiSeO (Fig. 3(d–f)) and blueP-ZrSO (Fig. 3(g–i)), and at the *K*-point of BZ for blueP-ZrSeO ((Fig. 3(j–l)), and at the *M*–*Γ*-point of BZ for blueP-HfSO (Fig. 3(m–o)) and for blueP-HfSeO (Fig. 3(p–r)) vdW heterostructures. The bandgap values are presented in Table 2, and are in the range of 0.88–1.94 eV and the average of the corresponding monolayers is in agreement with ref. 57, 58 and 62.

We have further investigated the weighted band structures presented in Fig. S5,† to elaborate the maximum contributions of atomic sites near the Fermi level. In the case of the blueP-TiSO (Fig. S5(a–c)†) vdW heterostructures, one can easily see that VBM(CBM) is mainly due to the *p*_*z*_(d_*xy*_) state of the blueP(TiSO) monolayer. This type of contribution of the states of different atoms of vdW heterostructures in the VBM and CBM indicates that type-II (staggered gap) band alignment, slows down charge recombination rate, revealing promising applications in optoelectronic, solar energy conversion and photocatalysis.^34,63,64^ These localized layers in VBM(CBM) potentially act as the electron acceptor(donor) in corresponding vdW heterostructures. Type-II band alignment is also observed in blueP-ZrSO (Fig. S5(g–i)†), blueP-ZrSeO (Fig. S5(j–l)†), blueP-HfSO (Fig. S5(m–o)†) and blueP-HfSO (Fig. S5(p–r)†) vdW heterostructures, while in ref. 65, type-II band alignment is reported in the case of direct bandgap semiconductors only. In the case of the blueP-TiSeO (Fig. S5(d–f)†) vdW heterostructure, VBM(CBM) is due to the d_*xz*_ state of TiSO monolayer type-I (straddling gap) band alignment, significant for designing light emitting diodes and laser devices.^66,67^ Similar results are also observed in BP-BSe vdW heterostructures.^68^

Generally, type-II band alignment facilitates the charge transfer,^69^ therefore using the charge density difference (*ρ* = *ρ*_blueP–XYO_ − *ρ*_blueP_ − *ρ*_XYO_) for all three blueP-TiSO vdW stacking heterostructures (see Fig. S6†) – where *ρ*_blueP–XYO_ represents the charge density of blueP–XYO vdW heterostructures, *ρ*_blueP_ is the charge density of the blueP monolayer and *ρ*_XYO_ is the charge density of the XYO monolayers – we have investigated the interlayer charge transfer. Electrons are transferred from blueP to XYO monolayers in all three of the blueP-TiSO, blueP-TiSeO, blueP-ZrSO, blueP-ZrSeO, blueP-HfSO and blueP-HfSeO vdW heterostructures. Charge redistribution is mainly establish in the interfacial region between the constituent layers of vdW heterostructures due to the electronegativity difference, and induces a built in electric field, hence separates the charge carriers in the corresponding layers. This indicates the weak interaction between blueP and XYO monolayers, which has also been demonstrated in MXY–MXY vdW heterostructures.^57^ The layer of the blueP(XYO) donate(accept) electrons represented by yellow(other colors) in Fig. S6,† leads to p(n)-doping in the corresponding monolayers.

We further perform the Bader charge analysis for the quantitative analysis of the transfer of electrons between the corresponding layers of the blueP-XYO (X = Ti, Zr, Hf; Y = S, Se) vdW heterostructures. Bader charge analysis shows that about 0.09*e* (stacking (a)), 0.088*e* (stacking (b)) and 0.096*e* (stacking (c)) are transferred from blueP to TiSO in the blueP-TiSO vdW heterostructure; 0.11*e* (stacking (a)), 0.0955*e* (stacking (b)) and 0.106*e* (stacking (c)) are transferred from blueP to TiSeO in the blueP-TiSeO vdW heterostructure; 0.063*e* (stacking (a)), 0.052*e* (stacking (b)), and 0.069*e* (stacking (c)) are transferred from blueP to ZrSO in the blueP-ZrSO vdW heterostructure; 0.135*e* (stacking (a)), 0.126*e* (stacking (b)), and 0.119*e* (stacking (c)) are transferred from blueP to ZrSeO in the blueP-ZrSeO vdW heterostructure; 0.05*e* (stacking (a)), 0.045*e* (stacking (b)), and 0.053*e* (stacking (c)) are transferred from blueP to HfSO in the blueP-HfSO vdW heterostructure; and 0.13*e* (stacking (a)), 0.10*e* (stacking (b)) and 0.18*e* (stacking (c)) are transferred from blueP to HfSeO in the blueP-HfSeO vdW heterostructure.

**Fig. 4:**
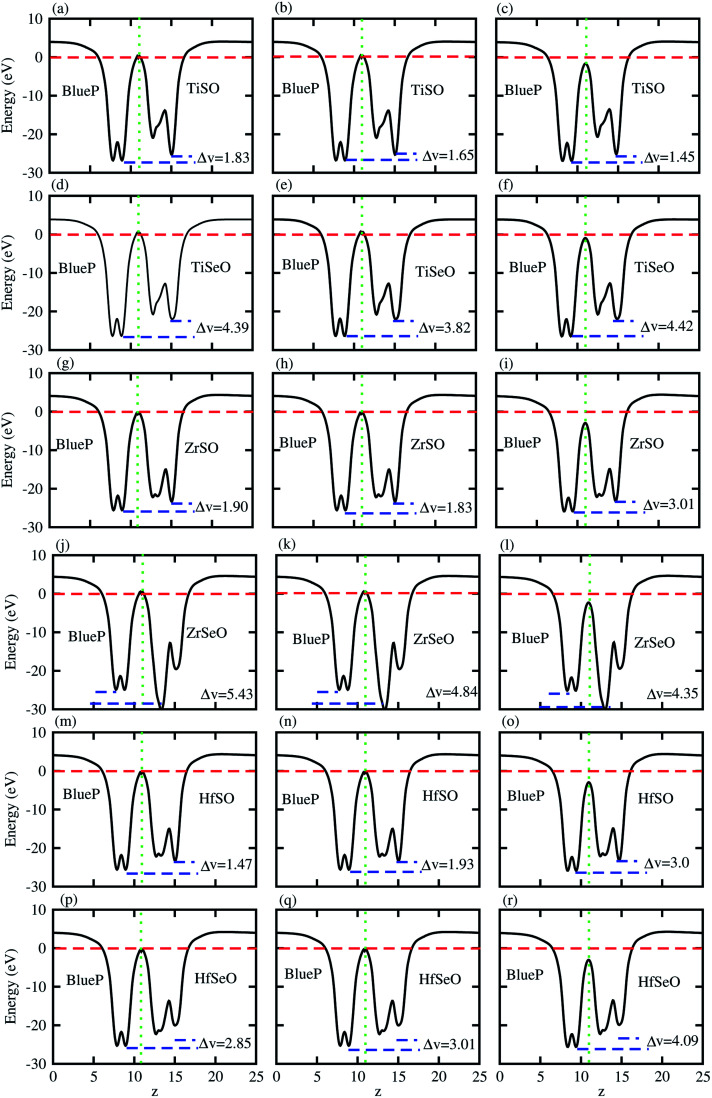
Averaged charge density difference of blueP-TiSO (a–c), blueP-TiSeO (d–f), blueP-ZrSO (g–i), blueP-ZrSeO (j–l), blueP-HfSO (m–o), and blueP-HfSeO (p–r), where the first column is for stacking-I, the second column is for stacking-II and the third column is for stacking-III of the corresponding vdW heterostructures.

The charge transfers are further confirmed by calculation of the average electrostatic potential of blueP-XYO (X = Ti, Zr, Hf: Y = S, Se) vdW heterostructure, see Fig. 4. The deeper potential of blueP compared to the XYO indicates the driving of electrons from the blueP to XYO layer. The potential drop across the bilayer, for the blueP-TiSO vdW heterostructure is 1.62 eV (stacking (a)), 1.47 eV (stacking (b)) and, 2.01 (stacking (c)); for the blueP-TiSeO vdW heterostructure it is 4.39 eV (stacking (a)), 3.82 eV (stacking (b)) and 4.42 eV (stacking (c)); for the blueP-ZrSO vdW heterostructure it is 1.90 eV (stacking (a)), 1.83 eV (stacking (b)) and 3.01 eV (stacking (c)); for the blueP-ZrSeO vdW heterostructure it is 5.43 eV (stacking (a)), 4.84 eV (stacking (b)) and 4.35 eV (stacking (c)); for the blueP-HfSO vdW heterostructure it is 2.36 eV (stacking (a)), 2.20 eV (stacking (b)) and 2.50 eV (stacking (c)); and for the blueP-HfSeO vdW heterostructure it is 2.90 eV (stacking (a)), 2.81 eV (stacking (b)) and 3.09 eV (stacking (c)), see Fig. 4 for details. This type of potential drop gives a strong electrostatic field between the layers with transport barriers, hence has an astonishing effect on the dynamics of the charge carrier. These results are in agreement with other vdW heterostructures based on Janus monolayers.^70–72^

At the surface, the direction of the charge flow is established by the work function (*ϕ*):^73,74^*ϕ* = *V*_∞_ − *E*_F_. Here *V*_∞_ is the electronic potential at a vacuum region far from the surface and *E*_F_ is the Fermi level, presented in Table 2. Calculated *ϕ* of the vdW heterostructures under study show the trend: blueP-ZrSO > blueP-ZrSeO > blueP-HfSO > blueP-HfSeO > blueP-TiSeO > blueP-TiSO, which is due to the lower ionization energy of the Ti-atom as compared to Hf and Zr-atoms. These values also confirm the transportation of electrons from blueP to XYO monolayers, which results in a built-in electric field at the interface, and hence reduces the recombination of photogenerated electrons and holes.

The effective mass of electrons and holes is calculated by fitting the conduction and valence band to a parabola, while implementing the deformation potential theory,^75^
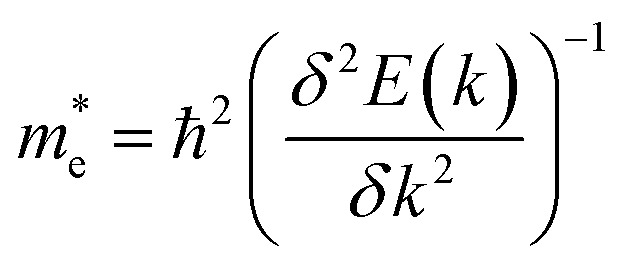
, where *k* is the wave vector and *E*(*k*) is the corresponding energy of *k*. In agreement with ref. 15, the calculated effective mass of electrons (holes) for TiSO is 1.23(0.41)*m*_e_, for TiSeO is 1.26(0.32)*m*_e_, for ZrSO is 2.53(0.61)*m*_e_, for ZrSeO is 1.67(0.40)*m*_e_, for HfSO is 1.90(0.58)*m*_e_ and for HfSeO is 1.30(0.28)*m*_e_. In the case of the vdW heterostructures, the calculated effective mass of electrons (holes) in: stacking (a) for blueP-TiSO is 1.37(0.56)*m*_e_, for blueP-TiSeO is 1.41(0.47)*m*_e_, for blueP-ZrSO is 2.61(0.53)*m*_e_, for blueP-ZrSeO is 1.80(0.55)*m*_e_, for blueHfSO is 2.12(0.69)*m*_e_ and for blueP-HfSeO is 1.43(0.33)*m*_e_; in stacking (b) for blueP-TiSO is 1.30(0.49)*m*_e_, for blueP-TiSeO is 1.52(0.56)*m*_e_, for blueP-ZrSO is 2.72(0.60)*m*_e_, for blueP-ZrSeO is 1.98(0.65), for blueHfSO is 2.10 (0.59) and for blueP-HfSeO is 1.37(0.31); in stacking (c) for blueP-TiSO is 1.24 (0.38), for blueP-TiSeO is 1.47(0.50), for blueP-ZrSO is 2.60(0.54), for blueP-ZrSeO is 1.90(0.61), for blueP-HfSO is 1.96(0.52) and for blueP-HfSeO is 1.30 (0.29). A greater transport of charge carriers follows owing to the smaller effective mass,^76^ desirable for competent electronic and optoelectronic devices. Therefore, from the calculated effective mass of electrons and holes, monolayers of TiSO, TiSeO and also their vdW heterostructures blueP-TiSO and blueP-TiSeO, are highly recommended for high performance device applications. Based on the higher effective mass of electrons over hole carriers, both monolayers and their vdW heterostructures are also recommended for utilization of electron/hole separation.

**Fig. 5:**
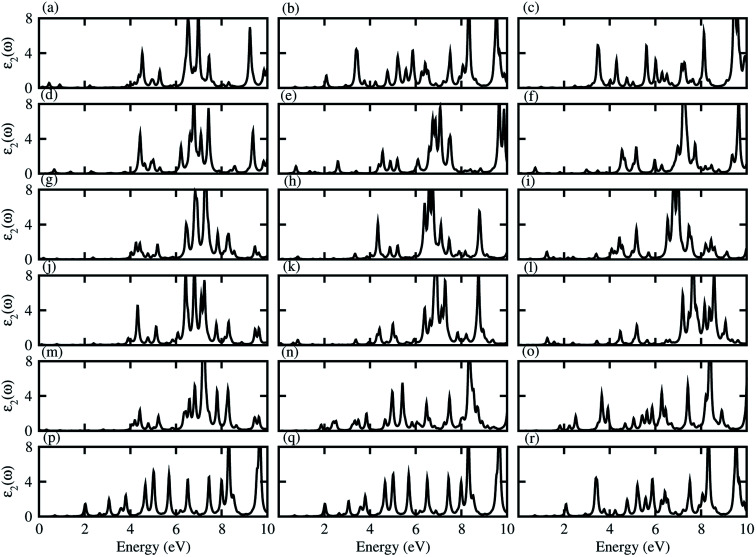
Imaginary part of the dielectric function of blueP-TiSO (a–c), blueP-TiSeO (d–f), blueP-ZrSO (g–i), blueP-ZrSeO (j–l), blueP-HfSO (m–o), and blueP-HfSeO (p–r), where the first column is for stacking-I, second column is for stacking-II and the third column is for stacking-III of the corresponding vdW heterostructures.

Dielectric function gives a connection between experiment and theory related to optical properties, hence is a prime parameter for the characterization of novel materials. The imaginary part of the dielectric function (*ε*_2_(*ω*)), presented in Fig. 5, shows that excitons dominate the optical transitions in the case of blueP, TiSO, TiSeO, ZrSO, ZrSeO, HfSO and HfSeO monolayers (see Fig. S7†), and their corresponding vdW heterostructures (see Fig. 5). The position of the excitonic peaks (with binding energy) appear in the absorption spectra at 3.7(0.93) eV for blueP, 3.8(1.62) eV for TiSO, 3.3(1.77) eV for TiSeO, 3.12(1.15) eV for ZrSO, 2.36(1.18) eV for ZrSeO, 3.19(1.22) eV for HfSO and 2.28(1.45) eV for HfSeO monolayers. In the case of the vdW heterostructures, these peaks appear in the absorption spectra of blueP-TiSO at 0.34–0.65 eV (stacking (a)), 1.05–2.1 eV (stacking (b)), and 0.95–1.95 eV (stacking (c)); blueP-TiSeO at 0.40–0.75 eV (stacking (a)), 0.37–2.2 eV (stacking (b)), and 0.36–2.06 eV (stacking (c)); blueP-ZrSO at 2.02–3.93 eV (stacking (a)), 0.39–3.86 eV (stacking (b)), and 0.59–3.76 eV (stacking (c)); blueP-ZrSeO at 0.29–3.98 eV (stacking (a)), 0.36–3.80 eV (stacking (b)), and 0.83–3.25 eV (stacking (c)); blueP-HfSO at 2.25–2.3 eV (stacking (a)), 1.63–1.72 eV (stacking (b)), and 1.52–1.60 eV (stacking (c)); and blueP-HfSeO at 1.35–1.86 eV (stacking (a)), 1.40–1.81 eV (stacking (b)), and 1.35–2.15 eV (stacking (c)). Increasing carrier density extends the exciton transition in the heterostructure with respect to the corresponding monolayers, which further broadens and red-shifts, while a systematic red shift of the excitonic peaks with heavier chalcogen atoms is also noticed. A similar trend is observed in Janus–Janus,^57^ ZnO-Janus^58^ and MoSe_2_/blue-phosphorene^59^ vdW heterostructures, suggesting that these vdW heterostructures provide control of exciton–phonon coupling on the nanoscale.^57,58^ The exciton binding energy (*E*_b_) is an important physical quantity to describe the optical characteristics of semiconductors, defined as: excitons estimated by the difference between the excitation energy and the quasi-particle energy difference.^60,61^ We have calculated the binding energies from the first peak to the imaginary part of the dielectric function. Excitonic binding energy in the case of blueP-TiSO is 0.6 eV (stacking (a)), 0.26 eV (stacking (b)), and 0.07 eV (stacking (c)); in the case of blueP-TiSeO is 0.57 eV (stacking (a)), 0.94 eV (stacking (b)), and 0.63 eV (stacking (c)); in the case of blueP-ZrSO is 0.53 eV (stacking (a)), 0.54 eV (stacking (b)), and 0.24 eV (stacking (c)); in the case of blueP-ZrSeO is 0.7 eV (stacking (a)), 0.67 eV (stacking (b)), and 0.09 eV (stacking (c)); in the case of blueP-HfSO is 0.31 eV (stacking (a)), 0.27 eV (stacking (b)), and 0.36 eV (stacking (c)); and in the case of blueP-HfSeO is 0.31 eV (stacking (a)), 0.39 eV (stacking (b)), and 0.38 eV (stacking (c)). The calculated exciton binding energy of both monolayers and their corresponding vdW heterostructures are in the range of previously calculated values for MX_2_ (M = Mo, W; X = S, Se, Te) monolayers and their vdW heterostructures in ref. 18.

A photocatalytic conversion of solar light into hydrogen, is an attractive technique for the production of clean and renewable energy device applications.^77^ In semiconductors, under irradiation of light, excited electrons(holes) in the conduction(valence) band get involved in hydrogen(oxygen) evolution reactions for the production of H_2_(O_2_) gas under; 
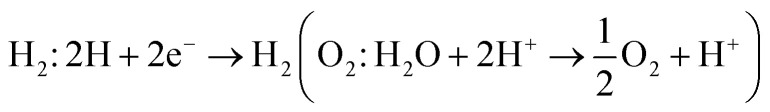
. Therefore, materials having a minimum bandgap of 1.23 eV with suitable ionization and electron affinity are promising for carrying out both reactions, hence are efficient to utilize solar light.^78,79^

**Fig. 6:**
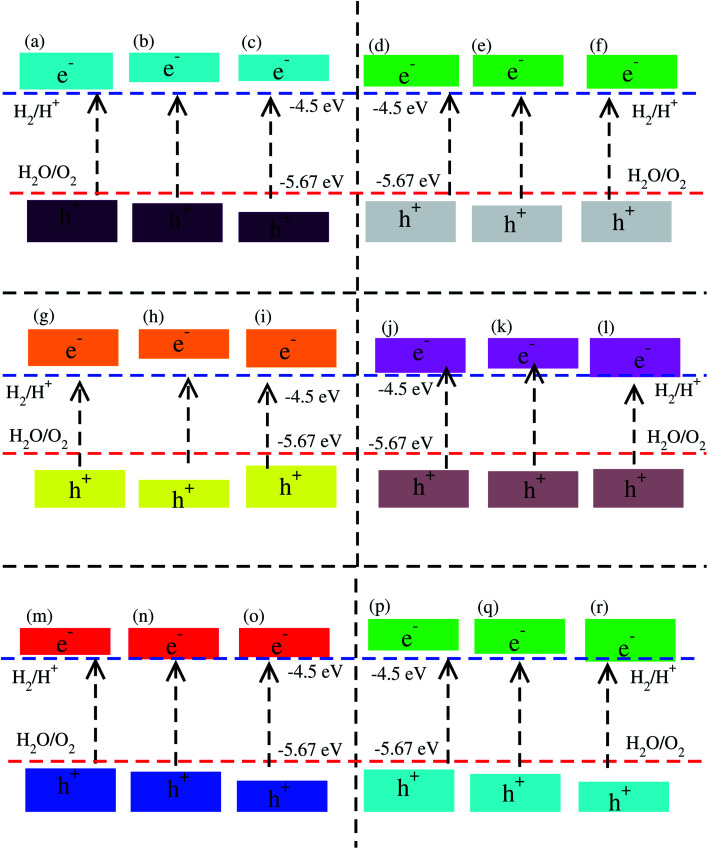
Valence band (*E*_VB_) and conduction band (*E*_CB_) edge alignment of blueP-TiSO (a–c), blueP-TiSeO (d–f), blueP-ZrSO (g–i), blueP-ZrSeO (j–l), blueP-HfSO (m–o), and blueP-HfSeO (p–r), where the first column is for stacking-I, the second column is for stacking-II and the third column is for stacking-III of the corresponding vdW heterostructures. The standard oxidation (−5.67 eV) and reduction (−4.44 eV) potentials for water splitting into O_2_ = H_2_O and H^+^ = H_2_, respectively.

Using Mulliken electronegativity,^80,81^ valence band edge (*E*_VB_) and conduction band edge (*E*_CB_) for the studied XYO (X = Ti, Zr, Hf: Y = S, Se) monolayers and their corresponding vdW heterostructures in all three possible stacks of blueP-TiSO, blueP-TiSeO, blueP-ZrSO, blueP-ZrSeO, blueP-HfSO, and blueP-HfSeO are calculated. The band edge positions in connection with water reduction(oxidation) potential levels are displayed, and the *E*_VB_(*E*_CB_) is set at 1.23(0) eV, equal to −5.67(−4.50) eV for aqua solution at pH = 0 (see Fig. S8† and Fig. 6). In the case of TiSO, ZrSO, ZrSeO and HfSO monolayers, the *E*_VB_ potential is more positive than O_2_/H_2_O (1.23 eV) and the *E*_CB_ potential is more negative than H^+^/H_2_, hence both *E*_VB_ and the *E*_CB_ edges achieve the energetically favorable positions and straddle the redox potentials, therefore, are suitable for water splitting at pH = 0; while for TiSeO and HfSeO monolayers, the *E*_VB_ potential is more positive than O_2_/H_2_O (1.23 eV) and showed a good response for reduction and failed to oxidise water at pH = 0 (see Fig. S6†). Furthermore, it is clear from Fig. 6 that all studied vdW heterostructures show energetically suitable positions for the *E*_VB_ band edges which are outside of the reduction potentials, and hence show a good response to produced O_2_, while the band edge position of blueP-TiSO (stacking (a), (b), and (c)), blueP-TiSeO (stacking (a), (b), and (c)), blueP-ZrSO (stacking (a), (b), and (c)), blueP-ZrSeO (stacking (a), (b)), blue-HfSO (stacking (a)) and blueP-HfSeO (stacking (a), (b)) are suitable *E*_VB_ band edges, and hence show an energetically good response to reduce H^+^ to H_2_. BlueP-HfSSe at pH = 0, fails to reduce H^+^ to H_2_ (See Fig. 6).

## 4. Conclusion

Using first-principles calculations, the geometry, electronic structure, optical and photocatalytic performance of blueP and XYO (X = Ti, Zr, Hf; Y = S, Se) monolayers and their corresponding vdW heterostructures in three possible stacking patterns are investigated. BlueP and XYO (X = Ti, Zr, Hf; Y = S, Se) monolayers are indirect bandgap semiconductors, while a tensile strain of 8(10)% leads to a transition of TiSeO(ZrSeO) monolayers from an indirect bandgap nature of 0.91(1.18) eV, to a direct bandgap nature of 1.30(1.61) eV. Based on the calculated binding energy and AIMD simulation, unstrained(strained) blueP and XYO (X = Ti, Zr, Hf; Y = S, Se) monolayers and their corresponding vdW heterostructures are found to be thermodynamically stable. Similar to the corresponding monolayers, blueP-XYO (X = Ti, Zr, Hf: Y = S, Se) vdW heterostructures in three possible stacking patterns are also found to be indirect bandgap semiconductors with a staggered gap band alignment, except for the blueP-TiSeO vdW heterostructure, which signifies a straddling gap band alignment. In the case of all three possible stacking patterns of the vdW heterostructure electrons, transfers occur from the blueP to XYO layer. Based on the higher effective mass of electrons over the hole carrier, both monolayers and their vdW heterostructures are recommended for utilization of electron/hole separation, while TiSO, TiSeO, blueP-TiSO and blueP-TiSeO are highly recommended for high performance device applications. Furthermore, absorption spectra show that the optical transitions are dominated by excitons for blueP and XYO (X = Ti, Zr, Hf; Y = S, Se) monolayers and their corresponding vdW heterostructures. Both *E*_VB_ and *E*_CB_ edges in TiSO, ZrSO, ZrSeO and HfSO monolayers achieve energetically favorable positions, and are therefore suitable for water splitting at pH = 0, while TiSeO and HfSeO monolayers showed a good response for reduction and fail to oxidise water. All studied vdW heterostructures show a good response to produced O_2_, while specific stacks are energetically favorable to reduce H^+^ to H_2_ at pH = 0.

## Conflicts of interest

There are no conflicts to declare.

## Supplementary Material

RA-011-D0RA10827H-s001
