# Supplementary material for: Stacking effects in van der Waals heterostructures of blueP and Janus XYO (X = Ti, Zr, Hf: Y = S, Se) monolayers
Source: RSC Adv. 2021 Mar 25;11(20):12189–99. doi: 10.1039/d0ra10827h (PMC8696924; doi:10.1039/d0ra10827h)
Supplement: RA-011-D0RA10827H-s001 [file RA-011-D0RA10827H-s001.pdf]

Electronic Supplementary Material (ESI) for RSC Advances.
This journal is © The Royal Society of Chemistry 2021

**Supplementary Material**

# Stacking Effects in van der Waals Heterostructures of BlueP and Janus XYO (X=Ti, Zr, Hf: Y=S, Se) monolayers

Qaisar Alam[1], M. Idrees[1], S. Muhammad[1], Chuong V. Nguyen[2], M. Shafiq[3], Y. Saeed[3], H. U. Din[3], B. Amin[3*]

[1]*Department of Physics, Hazara University, Mansehra 21300, Pakistan,*

[2]*Department of Materials Science and Engineering, Le Quy Don Technical University, Hanoi, Vietnam,*

[3]*Department of Physics, Abbottabad University of Science and Technology, Abbottabad, Pakistan.*

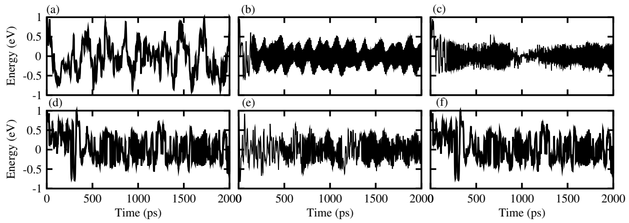


Fig.S 1: Thermal stabilities of strained (a) TiSO, (b) TiSeO, (c) ZrSO, (d) ZrSeO, (e) HfSO, and (f) HfSeO,

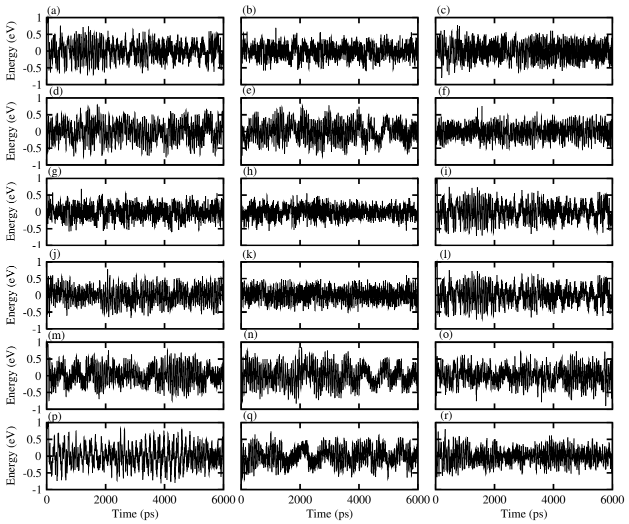


Fig.S 2: Thermal stabilities of blueP-TiSO ((a)-(c)), blueP-TiSeO ((d)-(f)), blueP-ZrSO ((g)-(i)), blueP-ZrSeO ((j)-(l)), blueP-HfSO ((m)-(o)), and blueP-HfSeO ((p)-(r)), where first column is for stacking-I, second column is for stacking-II and third column is for stacking-III of the corresponding vdW heterostructures

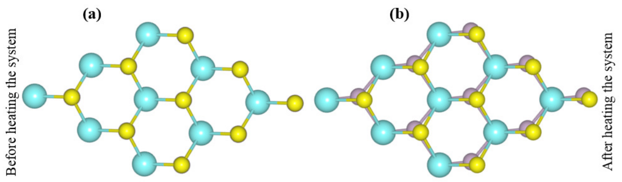


Fig.S 3: The atomic structure of the TiSeO monolayer (a) before and (b) after heating 6 ps, with no structure distortion.

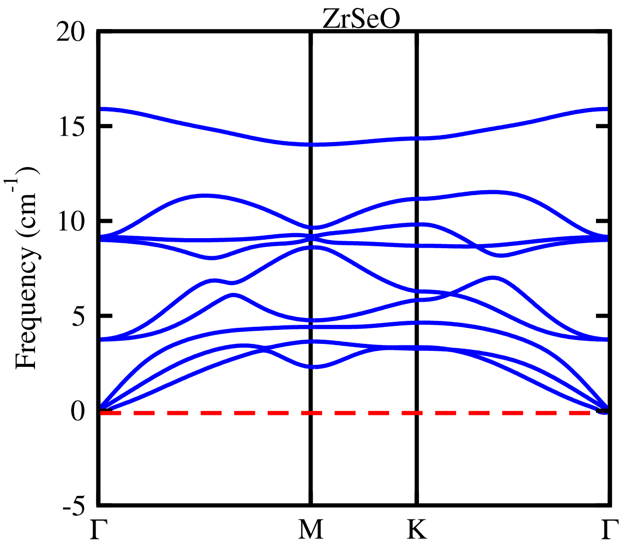


Fig.S 4: Phonon spectrum for 10% tensile strained ZrSSe.

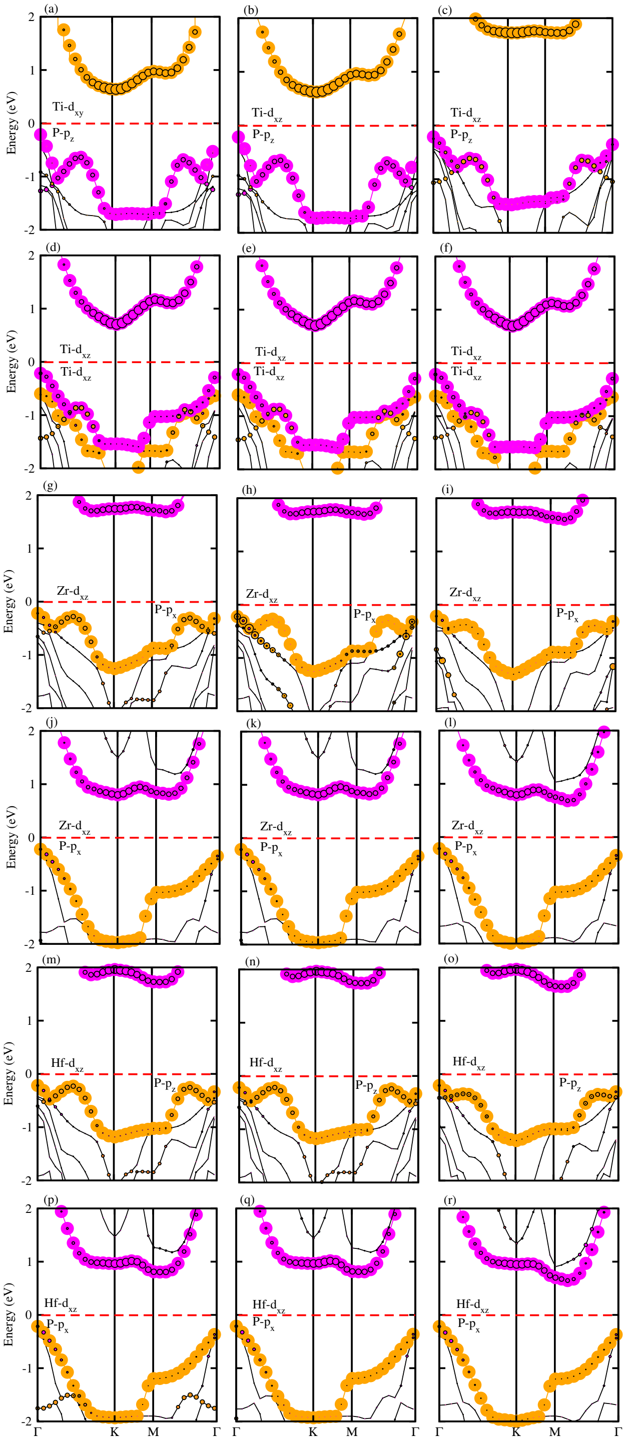


Fig.S 5: Weighted band structure of blueP-TiSO ((a)-(c)), blueP-TiSeO ((d)-(f)), blueP-ZrSO ((g)-(i)), blueP-ZrSeO ((j)-(l)), blueP-HfSO ((m)-(o)), and blueP-HfSeO ((p)-(r)), where first column is for stacking-I, second column is for stacking-II and third column is for stacking-III of the corresponding vdW heterostructures

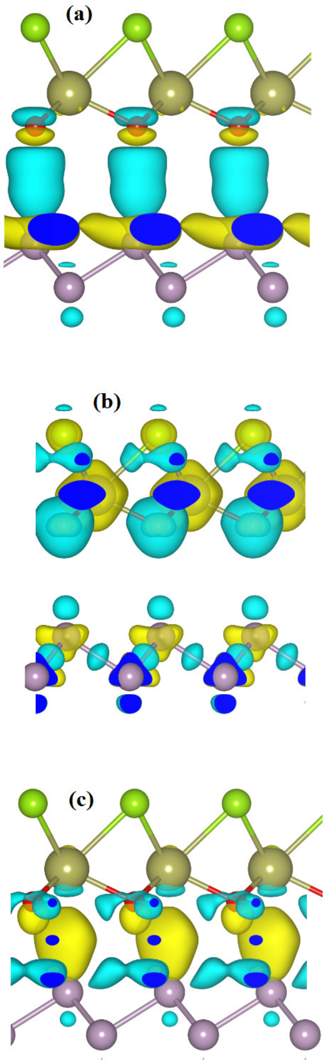


Fig.S 6: Charge density difference of the blueP-TiSO vdW heterostructure (for all three stakings), where the isovalue chosen to plot the isosurface is 0.001 eV A$^{-3}$

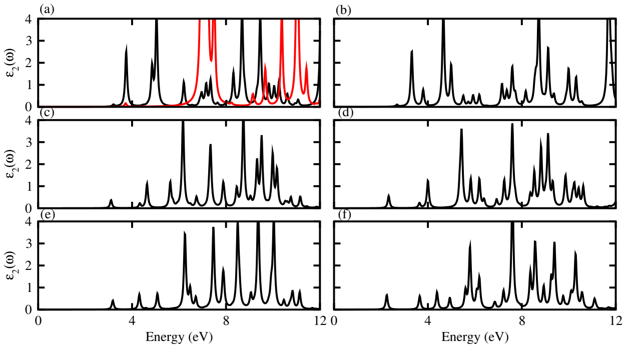


Fig.S 7: Imaginary part of dielectric function of (a) TiSO, (b) TiSeO, (c) ZrSO, (d) ZrSeO, (e) HfSO, and (f) HfSeO

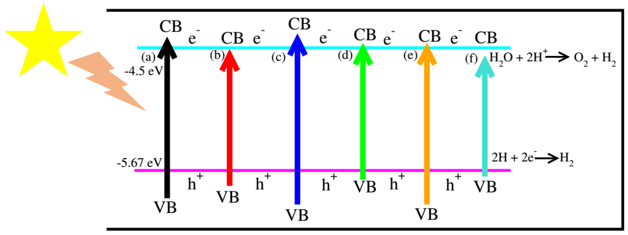


Fig.S 8: Valence band ($E_{VB}$) and conduction band ($E_{CB}$) edge alignment of (a) TiSO, (b) TiSeO, (c) ZrSO, (d) ZrSeO, (e) HfSO, and (f) HfSeO. The standard oxidation (-5.67 eV) and reduction (-4.44 eV) potentials for water splitting into $O_2 = H_2O$ and $H^+ = H_2$, respectively
